# Frameless Stereotactic Radiosurgery, a Feasible Alternative to the Frame-Based Technique for the Treatment of Refractory Trigeminal Neuralgia

**DOI:** 10.7759/cureus.571

**Published:** 2016-04-15

**Authors:** Andrew E Graff, Andrew S Thomas, Aaron D Reed, William K Skinner

**Affiliations:** 1 Radiation Oncology, Walter Reed National Military Medical Center

**Keywords:** stereotactic radiosurgery, frameless stereotactic radiosurgery, trigeminal neuralgia, refractory trigeminal neuralgia, facial pain

## Abstract

Classic trigeminal neuralgia (TN) causes severe facial pain. Several treatment options exist for classic TN refractory to medical therapy, including stereotactic radiosurgery (SRS). Most studies in the medical literature used a frame-based SRS technique. Improvements in linear accelerator-based treatment systems and image guidance have led to the use of frameless SRS as a safe and feasible alternative to the frame-based technique for the treatment of refractory TN. We present a case of refractory TN successfully treated with frameless SRS.

## Introduction

Trigeminal neuralgia (TN), also known as tic douloureux, is an uncommon condition known to cause severe facial pain [1]. The annual incidence of TN is low, 4 to 5 cases per 100,000 [2]. Risk factors for developing TN include female gender and increasing age [1-3]. The majority of TN cases are caused by compression of the trigeminal nerve root, most often from an aberrant loop of artery of vein [1, 4-6]. The trigeminal nerve--cranial nerve (CN) V--has three major branches (V1, Ophthalmic; V2, Maxillary; and V3, Mandibular), which provide sensation to the face. Additionally, motor supply to the muscle of mastication is provided by V3. Classic TN includes facial pain caused by vascular compression of CN V or due to idiopathic causes [7].

TN is characterized by paroxysmal episodes of severe pain lasting between a few seconds to two minutes in the distribution of at least one branch of CN V, most commonly V2, followed by V3 [1, 7]. The facial pain is most often unilateral [5]. Pharmacotherapy with carbamazepine is usually the first line of treatment for patients with classic TN [8]. Patients with classic TN refractory to medical therapy can be considered for surgical intervention, including rhizotomy, microvascular decompression (MVD), or stereotactic radiosurgery (SRS) [1, 8].

Most studies evaluating the use of SRS for TN involve gamma knife radiosurgery (GKRS), a frame-based treatment system [8-11]. GKRS has been shown to be safe and effective in treating patients with refractory TN [8-11]. Advancements in dedicated linear accelerator (D-LINAC) technology with its improvement in image guidance, has led to the popularity of frameless SRS, which is a truly non-invasive procedure [12]. Several studies have demonstrated that the use of frameless image-guided radiosurgery (IGRS) using a D-LINAC-based treatment system for refractory TN is an acceptable radiosurgical alternative to GKRS [12-14]. Herein, we present a case of refractory TN successfully treated with frameless SRS.

## Case presentation

An 82-year-old elderly female with a history of severe right-sided TN was previously treated unsuccessfully with numerous medications and percutaneous rhizotomy. She was referred to the radiation oncology clinic for consideration of SRS after neurosurgical evaluation.

The patient’s right-sided facial pain began initially in the 1980s, but she reported a long period of disease quiescence until symptoms recurred in 2007, one year after suffering a right cerebral hemispheric embolic stroke. At that time, her TN was treated with carbamazepine with a good initial response, but in 2010 this was discontinued due to an increasing dose requirement associated with the development of intolerable side effects. Multiple alternative medications were prescribed including gabapentin, Lyrica, fentanyl transdermal patches, and sumatriptan, without significant improvement of her pain.

In December 2014 and January 2015, she underwent percutaneous rhizotomy with radiofrequency ablation to her right trigeminal nerve, but despite these interventions, her facial pain persisted. In February 2015, she underwent brain magnetic resonance angiography/magnetic resonance imaging (MRA)/(MRI) to include her right internal auditory canal. Imaging demonstrated a right superior cerebellar artery (SCA) loop abutting CN V, causing a mass effect on the nerve near the root entry zone (REZ). The patient was subsequently referred to neurosurgery for possible surgical intervention. After neurosurgical evaluation, due to her age and multiple medical comorbidities, she was not considered an ideal surgical candidate for MVD and was referred to radiation oncology for consideration of SRS.

The patient was initially seen by Radiation Oncology in March 2015. During consultation, she described her facial pain as unilateral located on her right side and spanning from her lip to her temple region near the auricle. The pain episodes were characterized as sharp, intermittent, unpredictable, and severe. On average, her pain was 6/10 with breakthrough periods of 8/10. The pain was associated with chewing/biting, swallowing, light touch, and palpation of her right eyelid or nose. She reported that her symptoms were significantly affecting her quality of life. Her medication regimen consisted of Lyrica and Fentanyl patches, with hydromorphone for severe discomfort. On physical examination, sensory abnormalities were noted, specifically tenderness to light palpation on the right side of her face. After her assessment, she was considered to be a good candidate for SRS, especially given her history of classic TN refractory to medical therapy and her comorbidities.

**Figure 1:**
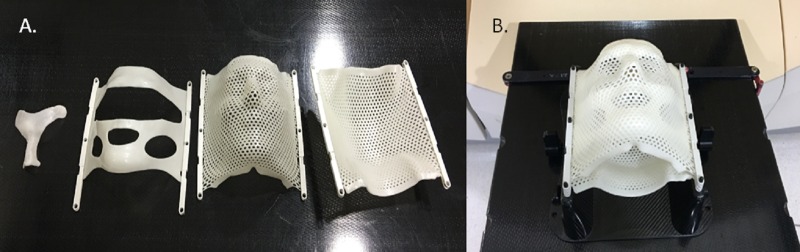
Custom SRS Mask. The image demonstrates the individual components to the SRS mask (A) and the assembled SRS mask (B).

In July 2015, the patient was simulated for treatment. Computed tomography (CT) was performed using .75 mm thick slices. At that time, a custom fitted SRS immobilization mask was created (Figure 1). Conical collimator planning was utilized using Brainlab iPlan RT planning software, which utilizes a pencil beam algorithm. An MRI was also performed prior to treatment and fused with the CT scan.

**Figure 2:**
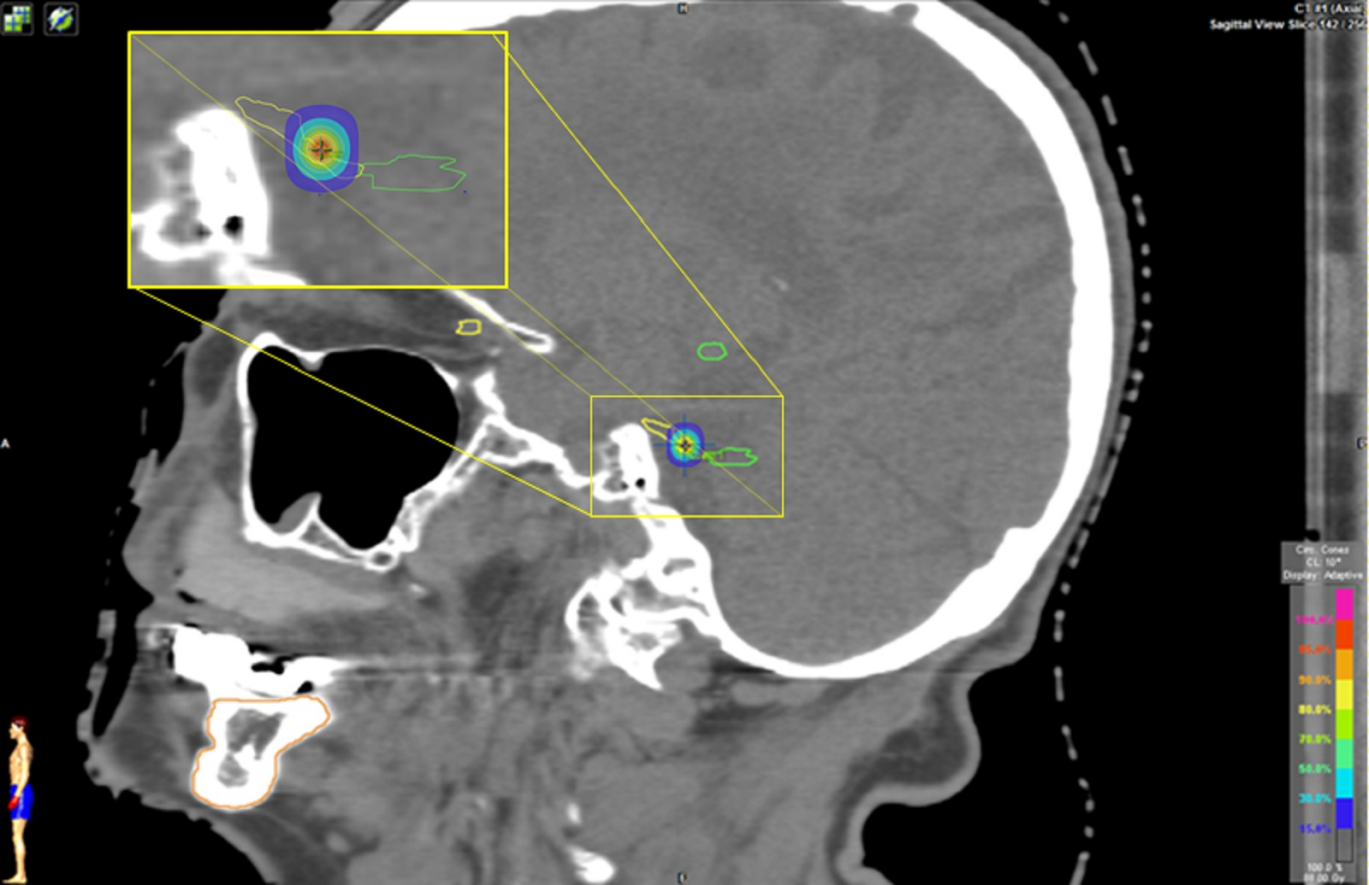
Sagittal Head CT. The image demonstrates targeted trigeminal nerve (contoured yellow) near its root entry into the brainstem (contoured green).

In August 2015, she underwent frameless cone collimator radiosurgery targeting the right trigeminal nerve, specifically the dorsal root entry zone (DREZ) (Figure 2).

**Figure 3:**
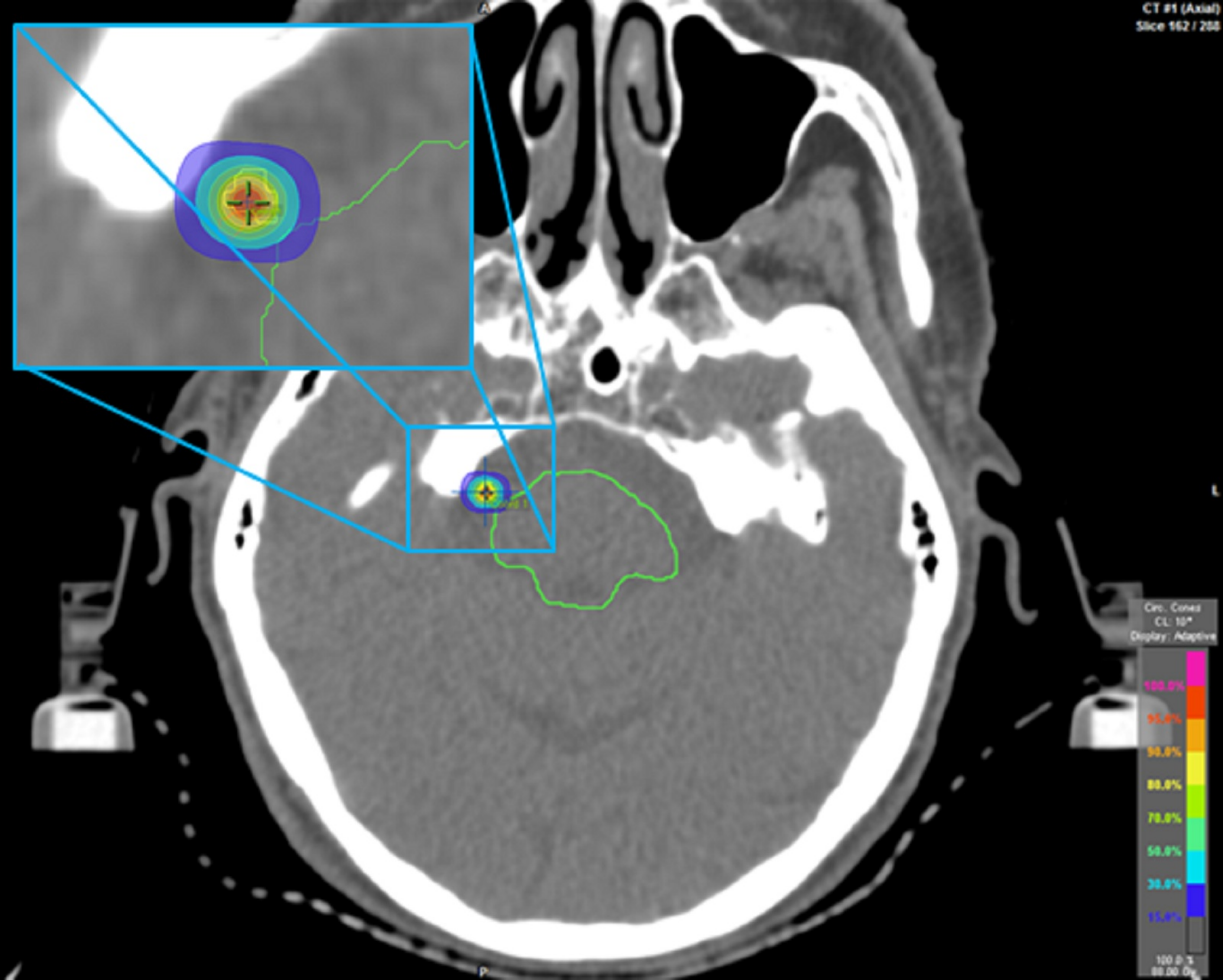
Axial Head CT. The image demonstrates the SRS treatment isocenter with 30% IDL abutting the brainstem (contoured green).

Prior to receiving SRS, she was premedicated with 6 mg of oral Decadron. She was set up on the treatment table in the supine position, and her custom immobilization mask was applied (Figure 1). She subsequently received 88 Gy prescribed to a single point dose with the 30% isodose line (IDL) abutting the brainstem (Figure 3). A volume of .019 cc of the nerve received at least 54 Gy.

Delivery of her radiation was completed using 6 MV photons on Varian’s TrueBeam, a D-LINAC treatment system. Seven arcs (SAD setup) were used during treatment totaling ~21,000 MUs at a 1,200 MU/min rate (couch angles: 0, 25, 50, 75, 280, 310, and 335 degrees). A 4 mm cone shaped the beam leaving the accelerator. Image guidance was performed using the Brainlab Novalis ExacTrac® system, which utilizes orthogonal x-rays to verify positioning at each treatment position. Her treatment was delivered over a period of one hour. Overall, the patient tolerated the procedure well without complaints. No anxiolytics were required during treatment.

At her two-month follow-up appointment, she reported that her facial pain had remitted within one week of treatment. She denied any facial pain, dry or painful eyes, and no sensation changes in the CN V region. The physical examination was normal with no CN deficits noted. She continues to report no facial pain without the need for pain medication six months after her treatment. A follow-up brain MRI is pending.

## Discussion

Multiple modalities exist for the treatment of refractory TN, among them include SRS and MVD. When comparing SRS and MVD, SRS has been shown to be just as effective at relieving pain long-term and is associated with lower rates of complications that arise from older age [15]. Stereotactic radiosurgery is believed to be a better treatment modality in the elderly than MVD due to the lower complication rate [15]. Most of the evidence evaluating the safety and efficacy of SRS treatment for TN involves the use of frame-based GKRS [9-10]. Technological advancements of D-LINAC treatment systems with image-guidance radiation therapy (IGRT) have translated into an increased popularity in employing frameless SRS treatment platforms due to their non-invasiveness and tolerability [12, 14]. Phantom studies have demonstrated that the frameless technique using the Novalis ExacTrac system is comparable in accuracy to the frame-based approach [16]. Retrospective studies have also demonstrated that D-LINAC-based frameless SRS is a suitable alternative to frame-based SRS, but no prospective or randomized controlled trials have been published to date [12, 14].

The etiology of TN is most often caused by the compression of the nerve root, usually due to a vascular source [1, 4, 6]. The MRI study was helpful in our case in identifying a vascular source causing nerve compression, but this is not always the case. Most MRI studies have been inconsistent in providing clear evidence to support their usefulness in identifying vascular causes of nerve compression. The studies have demonstrated their ability to identify structural causes (i.e., tumors, multiple sclerosis) and thus are recommended in the assessment and work-up in patients with TN [8, 17].

Determining the target treatment site and how much area to cover in the radiation treatment field is important to consider, both for symptom relief and to understand potential complications. The location of nerve compression is usually within millimeters of where the nerve enters the pons; this area is also called the REZ [1, 4, 6]. Several studies have shown a correlation between improved pain relief and the closeness of the treatment site to the REZ, but at a cost of increased facial numbness [14, 18-19]. These findings suggest that the REZ might be the optimal target for SRS. One randomized study compared two different treatment volumes, a single isocenter plan versus a two isocenter plan encompassing more of the trigeminal nerve. This study found no significant difference in pain relief, but an increased incidence in facial numbness in the larger treatment volume cohort [11].

The treatment isocenter is usually based on the IDL touching the brainstem [20]. Several definitions exist when describing isocenter based on IDLs for the treatment of TN, ranging from 20% IDL to 50% IDL touching the pons, tangential surface of the pons, brainstem, or adjacent to the region where the trigeminal nerve enters the brainstem (REZ) [20]. In our case, the isocenter was based off of the 30% prescribed IDL touching the brainstem (Figure 3). This technique has been described and used by other institutions [14, 21]. The use of conical collimator planning was utilized, which provides a steep dose gradient and falloff due to its conical design (Figures 2, 3). The steep dose gradient helps to minimize dose to surrounding normal tissue and organs at risk.

**Figure 4:**
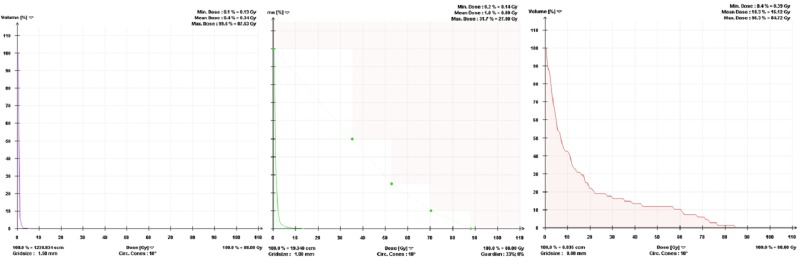
Dose Volume Histograms (DVHs). A. Whole Brain B. Brainstem C. Involved Loop of the Right SCA. The image demonstrates DVHs for the whole brain (A, solid purple line), brainstem (B, solid green line), and the involved SCA (C, solid red line).

The major organ at risk when targeting the DREZ is the brainstem. The treatment was prescribed to 88 Gy at the isocenter of the beams, 4.3 mm away from the brainstem and nerve root. The 75 Gy isodose line was only 2.4 mm away from the isocenter, with the brainstem itself receiving a maximum point dose of 27.9 Gy (Figure 4). Additionally, the mean dose to the brainstem was less than 1 Gy.

Other intracranial organs at risk received negligible doses (i.e., whole brain, optic chiasm, optic nerve, cochlea, and lens). The ideal treatment dose has yet to be determined, but most studies have used a range from 70 Gy to 90 Gy. Single institution studies have demonstrated high doses up to 90 Gy are acceptable due to the lack of major complications [14, 21-22]. Higher doses of radiation have also been associated with improved pain relief and a more durable response, but an increase in facial numbness was also seen [14].

Overall, SRS for refractory TN is a well-tolerated non-invasive procedure with good outcomes. The most commonly reported side effect is facial numbness, which is usually not bothersome in the majority of cases [14, 19-20]. As discussed above, facial numbness appears to be correlated with the distance from REZ, volume treated, and dose [11, 14, 18-19]. Other potential side-effects include eye irritation/dryness. Major side effects are much more commonly seen in other treatment techniques; these side effects include facial paresis, anesthesia dolorosa, subarachnoid hemorrhage, keratitis, balance problems, decreased hearing, or meningitis [20].

## Conclusions

Frameless SRS using a D-LINAC based system with modern IGRT is a safe and effective alternative to frame-based SRS for the treatment of classic TN refractory to medical therapy or surgery. Additional studies and long-term follow-up are needed to identify optimal dose and treatment target volume.

"The views expressed in this case report are those of the authors and do not reflect the official policy of the Department of Army/Navy/Air Force, Department of Defense, or U.S. Government."
